# Vascular Alterations Underlie Developmental Problems Manifested in Cloned Cattle before or after Birth

**DOI:** 10.1371/journal.pone.0106663

**Published:** 2015-01-13

**Authors:** Paulo Cesar Maiorka, Phelipe Oliveira Favaron, Andrea Maria Mess, Caio Rodrigues dos Santos, Miryan Lanca Alberto, Flavio Vieira Meirelles, Maria Angelica Miglino

**Affiliations:** 1 Department of Pathology, School of Veterinary Medicine, University of São Paulo, São Paulo, Brazil; 2 Department of Surgery, School of Veterinary Medicine, University of São Paulo, São Paulo, Brazil; 3 Faculty of Animal Sciences and Food Engineering, University of São Paulo, Pirassununga, Brazil; Osaka University Graduate School of Medicine, JAPAN

## Abstract

Although assisted reproductive techniques are commonly applied in humans and animals, they are frequently associated with major developmental deficits and reduced viability. To explore abnormalities associated with cloning or nuclear transfer (NT) as the most invasive of these methods, we used a bovine model to characterize abnormalities. Detailed necropsy examinations were done on 13 calves that died soon after birth; in addition, we included data from embryos and fetuses (produced by NT) that terminated prematurely. Bovine clones that survived until the neonatal period differed quantitatively and qualitatively from *in-vivo*-derived cattle. Although alterations affected a variety of organs (e.g. heart, lung and liver), there was a clear association with abberant vascular developmental during the early intrauterine phase. Therefore, we concluded that vascular problems were key alterations induced by cloning (presumably via epigenetic modifications).

## Introduction

Various assisted reproductive techniques (ART) are commonly used in human and large animal reproduction [1,2]. Several million children have been born produced via *in-vitro* fertilization (IVF) and other methods, providing promising results for individuals with reduced fertility [3]. In domestic animals, artificial insemination (AI) is widely used for genetic improvement, including sport horses as well as large-scale food production (cattle and swine). Furthermore, *in vitro* production (IVP) and transfer of bovine embryos is common. Finally, more invasive methods, including cloning or nuclear transfer (NT), are also used [4,5].

Reproductive techniques are also important for species preservation or gene banking [6]. However, there are limitations affecting the health of the offspring during pregnancy and postnatal life [7,8], many of which have not been thoroughly characterized. Animal models are essential in this regard, because ethical and medical considerations limit study in humans. The bovine model is valuable, due to its long gestation (similar to humans), well-developed newborns, and functional ART, including cloning [9]. Thus, detailed study of cloning (as the most invasive ART) should provide insights into developmental abnormalities caused by ART, both as a model for application of these technologies in humans and directly for livestock production [10]. Despite many years of experience in bovine cloning [9,11–13], efficiency remains very low (∼5% of pregnancies are maintained until term) and there is high postnatal mortality [9,14,15]. These development disturbances have been attributed to artificial treatments of germ cells and embryos, altered patterns of gene expression, and epigenetic disturbances [9,16–19]. Moreover, there are indications of severe alterations of the placental and yolk sac systems and their vasculature [16,20–24], as well as cardiovascular abnormalities [24–28]. In addition, perturbed immune system functions have also been reported [29,30], as well as increased body size (large offspring syndrome or gigantism), and multiple organ defects, most of which have only been superficially described [11,12,25,26,31–34]. In that regard, a comprehensive examination to characterize pathology has only been done on a very limited number of newborn clones [25,32]. Therefore, comprehensive pathological investigations are necessary to adequately characterize developmental abnormalities. In the present study, we investigated calves from NT bovine pregnancies that were born alive and died early in the neonatal period, focusing on qualitative aspects of gross pathology and histopathology. To complete previous work on early embryonic and fetal development [24], we included material from terminated NT pregnancies. Pathological data on other cloned animals including the sheep, swine and mice [35–37] were consulted for comparative aspects.

## Material and Methods

### Ethics statement

The research was approved by the Ethical Committee of the School of Veterinary Medicine and Animal Science of University of Sao Paulo, Brazil (Protocol 1393/2008).

### Nuclear transfer

The nuclear transfer and production of bovine clones followed established protocols [38,39]. Briefly, donor cells were prepared with serum starvation (DMEM.0.5% FBS) for 48 h before each round of micromanipulation. Oocytes aspirated from ovarian antral follicles (3–6mm in diameter) were selected (based on morphology) and matured *in vitro* for 18 h. Thereafter, oocytes were denuded and selected for extrusion of the first polar body and the donor cell was placed into the perivitelline space of each enucleated oocyte. Somatic cell nuclear transfer couplets were electrically fused, chemically activated, and cultured for 7 d. For recipients, healthy Nelore or zebu (*Bos indicus*) or cross-breed (*B. indicus* × *B. taurus*) heifers, 18–26 mo old, were used as embryo recipients. Without regard to the stage of their estrous cycle, heifers received a 3 mg norgestomet implant (Crestar, Intervet, Boxmeer, The Netherlands) placed SQ in the ear, plus 2 mg of estradiol benzoate (EB; Estrogin, Farmavet, Sao Paulo-SP, Brazil) given im. After 8 d, the implant was removed and 150 μg of d-cloprostenol (Preloban, Intervet) was given im, and 2 d later, embryos were nonsurgically transferred to the uterine horn ipsilateral to the ovary containing a corpus luteum. All recipients were kept on pasture (*ad libitum* access to mineral salt and water) at the Tambaú farm located near Tambaú, SP, Brazil. Pregnancy status was determined by transrectal ultrasonography approximately 28–30 d after embryos were transferred.

### Sample collection of prenatal stages

Studies on embryo development were supplemented with 6 embryos and fetuses, ranging from 32 to 70 d, with varying crown-rump-lengths (CRL). Tissues were prepared for histology and transmission electron microscopy (TEM), following previous protocols used in our laboratory [24,40].

### Sample collection of postnatal stages

In total, our samples were comprised of 13 neonatal calves from pregnancies that were carried to term, but died soon after birth, despite being treated with surfactant, antibiotics and anti-inflammatory drugs [41–43], were received for necropsy at the Department of Pathology, FMVZ-USP. Individual and biometric data are shown (Table 1). Samples were collected from all organs, fixed in 10% buffered formalin, and routinely processed for histopathology (embedded in paraffin wax, sectioned at 5 μm and stained with hematoxylin and eosin). For immunohistochemical analyses, a modified avidin-biotin peroxidase complex (ABC) amplification and detection system was used. After dewaxing and dehydration, antigen retrieval was done in citrate buffer (pH 6.0), by heating in a microwave oven (750 W) for 20 min). Thereafter, samples were incubated in 3% H2O2 for 10 min and blocked in a 2% solution of skim milk powder for 20 min. Sections were incubated overnight with primary antibody against CD3 (polyclonal, rabbit anti-human A0452, Dako Corporation, Carpinteria, CA, USA, 1:100 dilution) following Beltrão-Braga et al. [44].

**Table 1:** Data for cloned bovine fetuses, including sex (F = female and M = male), duration of post-natal survival (d = days and h = hours), body weight and weight of internal organs.

**No.**	**Sex**	**Surviving period**	**Body weight (kg)**	**Internal organs (g)**							
				**Thymus**	**Right lung**	**Left lung**	**Heart**	**Right kidney**	**Left kidney**	**Thyroid**	**Spleen**
1	F	15 d	53.3	30	620	475	513	26	72	646	6
2	F	5 d	52.5	25	943	505	433	173.68	153.18	1110	9.5
3	M	3 d	13.2	21	110	117	137	37	29	430	5
4	M	1 d	52.7	24	630	325	490	310	270	2600	12
5	M	4 h	51.2	55	525	450	450	255	263	1308	13
6	F	1 d	51.5	99	515	325	735	735	108	1620	7
7	F	½ h	62.0	45	460	515	743	743	177	1228	4
8	F	7 d	57.1	11	600	962	431	431	115	881	12
9	F	9 d	55.1	20	634	320	291	189	137	1650	9
10	M	2 d	45.9	25	1020	710	480	215	275	1775	12
11	M	20 d	60.2	35	769	517	436	137	144	975	13
12	F	9 d	47.1	80	630	511	405	168	164	1105	14
13	M	3 d	51.4	45.5	471	514	742	740	171	1180	8

## Results

### Morphopathology of embryos and fetuses

**Figure 1:**
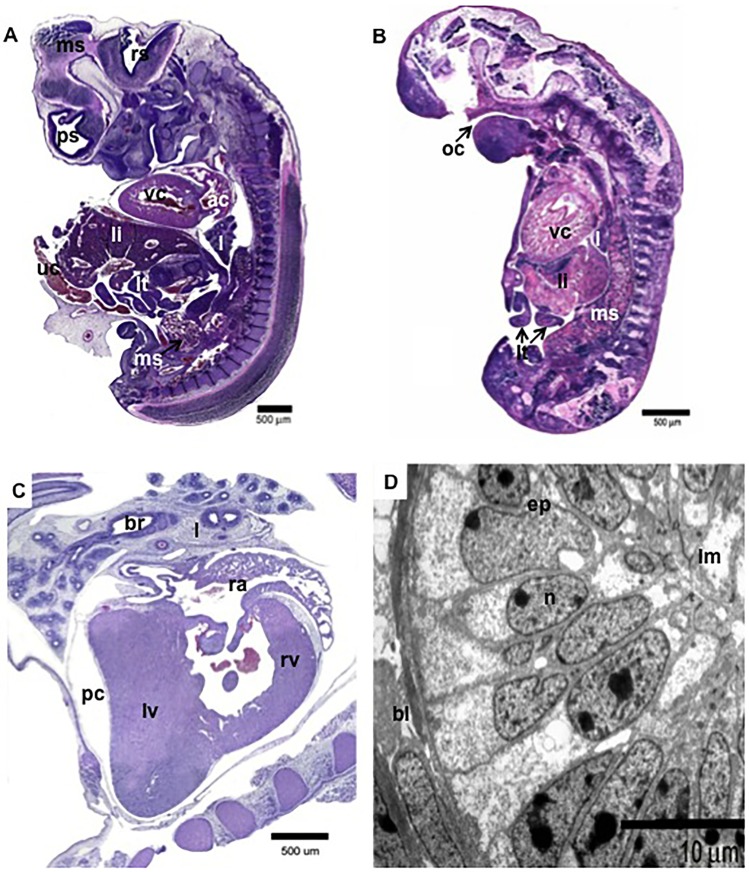
Morphology of cloned bovine embryos and fetuses. **(A):** Histology of 43 d old embryo (CRL, 27.5 mm). **(B):** Histology of 70-day fetus (13mm). **(C):** Histology of heart of a 68-d fetus (CRL, 53.3 mm). **(D):** TEM of a lung bronchus at 68 d. Shown are: prosencephalon (pc), mesencephalon (mc), rhombencephalon (rc), ventricular chamber (vc), atrial chamber (ac), lung (l), liver (li), mesonephros (ms), umbilical cord (uc), oral cavity (oc), intestine loops (it), pericardial cavity (pc), left ventricle (lv), right ventricle (rv), right atrium (ra), bronchus (br), epithelium (ep), nucleus (n), lumen (lm), and basal lamina (bl).

The 43-d embryo (27.5 mm CRL) had well-differentiated neurological compartments, a well-developed liver, and a heart with atrial and ventricular differentiation (Fig. 1A). Fetuses of 50 and 68 d had all their major organs present. However, a 70-d old fetus was very small (13 mm CRL); it had a rudimentary liver, lungs and other internal organs, but the heart was large (Fig. 1B). In particular, the heart musculature was prominent, round and had loose muscle fibers, with incomplete separation of the chambers (Fig. 1B). In contrast, a fetus of 68 d (53.3 mm CRL) had more normal development, including a developing heart with both atrial and ventricular chambers with a compact muscle fiber structure (Fig. 1C). However, lung bronchi had signs of edema (Fig. 1D).

### Pathology of postnatal calves

**Figure 2:**
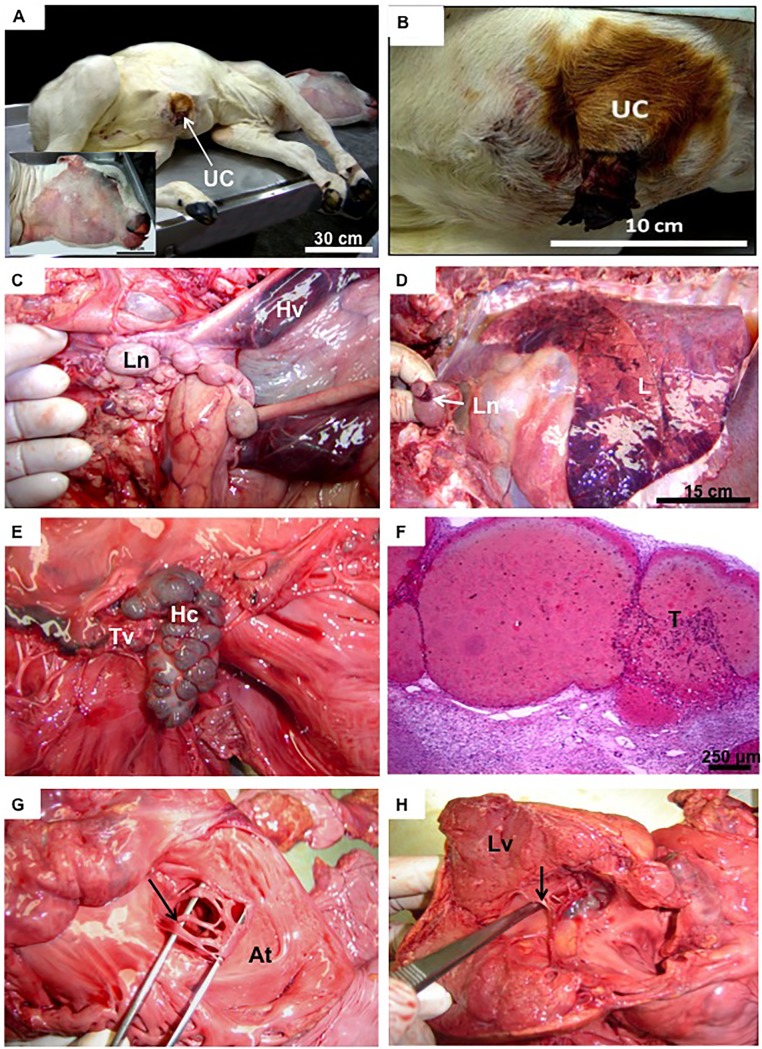
Cloned newborn calf with greatest body weight. **(A):** Clone No. 7, female, birth weight of 62.0 kg, delivered by Ceasarian section, but died within 0.5 h due to cardio-respiratory insufficiency. UC = umbilical cord. **(B and C):** Detail of the enlarged umbilical cord vessels, with lymphonoid swelling (Ln) and hematoma in the umbilical veins (Hv). **(D):** Lung (L) congestion with enlarged lymph node (Ln). **(E):** Hematic cyst (Hc) within tricuspid valve (Tv). **(F):** Histology of hematic cyst with organized thrombus (T). **(G):** Atrium (At) with incomplete foramen ovale or inter-atrial communication (arrow). **(H):** Left ventricle (Lv) with hypertrophy and interventricular communication (arrow).

**Figure 3:**
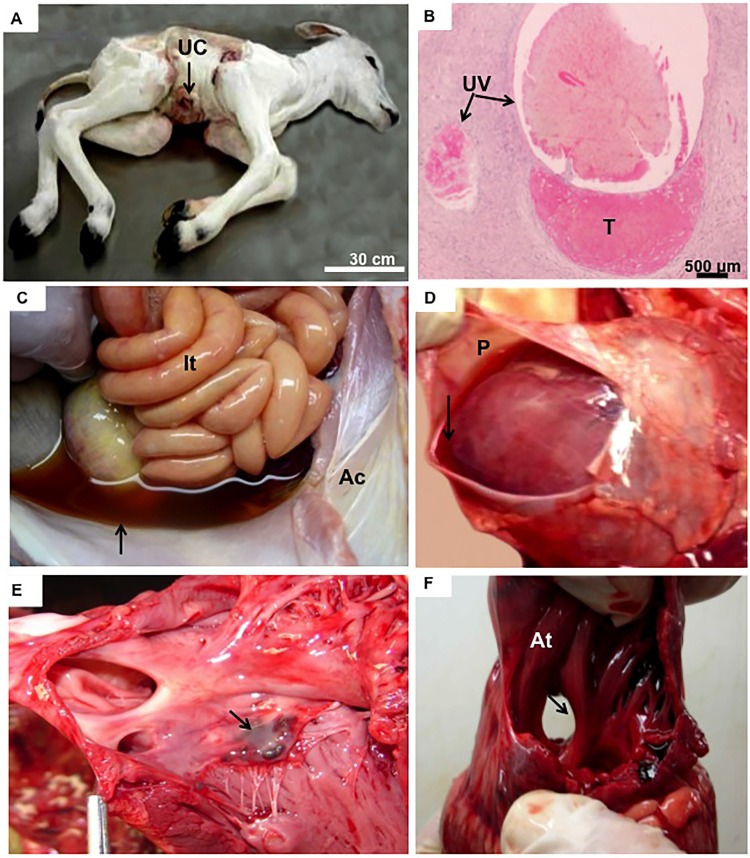
Cloned newborn calf with lowest body weight. **(A):** Clone No. 3, male, that died 3 days after birth with an enlarged umbilical cord (UC). **(B):** Histology of umbilical cord showing the umbilical veins (UV) and thrombus (T). **(C):** Abdominal cavity with intestine (It) and ascites (Ac). Note the presence of liquid inside the cavity (arrow). **(D):** Hydropericardium (P) filled with transudate (arrow). **(E):** Small hematic cyst (arrow) in tricuspid valve. **(F):** Atrium (At) with patent foramen ovale (arrow).

Body weight was outside the normal range in 92.3% of term calves that survived 1 to 20 d after birth (Table 1). Body weight was apparently not related to survival time after birth (Table 1) or to the severity of malformations. The most common abnormality was gigantism, with a maximum of 62 kg in an individual that died half an hour after birth (Fig. 2A–H). Also, birthweight of one calf that survived 20 d was ∼60 kg, whereas most calves had a birthweight between 45 and 55 kg (Table 1). In contrast, one calf was only 13 kg at 3 d of age (Table 1, Fig. 3A-F), and approximately the same weight at birth (F.V. Meirelles, pers. observation). Biometrics of the internal organs also resulted in a very broad range for all organs, likewise independent of survival rates and other parameters (Table 1, see below). Although these offspring were a single breed and all were produced using an identical cloning technique, there was an enormous range in size and weight of internal organs (Table 2).

**Table 2:** Weights of internal organs from cloned bovine fetuses.

**Organ**	**Minimum weight (g)**	**Maximum weight (g)**	**Average**	**Variation**
Thymus	11	99	32	800
Right lung	110	1020	621.3	827
Left lung	117	962	477.6	722
Heart	137	743	386.7	442
Right kidney	29	275	285	848
Left kidney	26	743	159	2757
Liver	430	2600	1277	504
Thyroid	4	14	9.7	250
Spleen	14	375	93.8	2578

**Figure 4:**
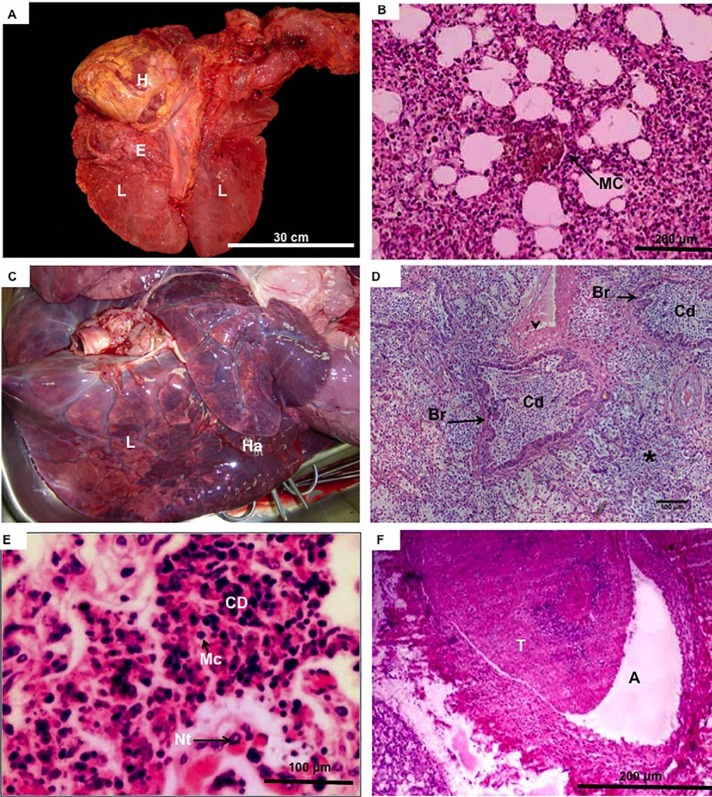
Details regarding internal organs of cloned bovine fetuese. **(A and B):** Clone No. 7, showing gross morphology of the lungs (L) with congestion and areas of emphysema (E), near overlarge heart (H), and histological evidence of meconium aspiration (MC). **(C–F):** Clone No. 1, female, that survived 15 d. **(C):** Lung (L) with aseptic pneumonia showing severe congestion, edema and areas of red hepatization (Ha). **(D):** Lung histology, with severe congestion showing cellular debris (Cd) fulfilling both alveolar (*) and bronchiolar spaces (Br). Note the fibrotic areas surrounding the vessels (arrowhead). **(E):** Detail of the areas of cellular debris (CD) with minimal inflammatory cells, such as macrophages (Mc) and neutrophil (Nt) and lack of bacteria. **(F):** Thrombus (T) in pulmonary artery (A).

Cyanotic mucous membranes and red-tinged liquid at the nostrils were common (70%). Umbilical cord swellings and enlarged umbilical vessels (Figs. 2A–B, 3A–B) were present in all calves, in addition to hematomas within the vessels (Fig. 2C). Some animals (30%) had anasarca (Fig. 2A), i.e. severe edema inside the body cavity (Fig. 3C). In most calves, there were large quantities of peritoneal effusion, with a brown tinge (Fig. 3C). Thoracic cavities had serous effusion with red liquid that included traces of fibrin (serofibrinoushydrothorax). There was commonly effusion in pericardial cavities (70%) (Fig. 3D). The surface of the lung lobes had mild adhesions and lymphadenomegaly of mesenteric lymph nodes in the thorax (Fig. 2C,D). Areas of emphysema distributed diffusely throughout the lung were common, especially in animals that died within the first 24 h (Fig. 4A–C), but were less pronounced in those that survived longer (Fig. 4B,D). Bilateral atelectases was common (70%) in calves that died within the first day, in addition to severe edema and meconium aspiration (Fig. 4C). Moreover, pulmonary congestions were present in all calves, usually distributed throughout the entire parenchyma, but more pronounced in dependent areas (Fig. 2D,4B). Mild to moderate pulmonary edema with the externalization of reddish (sero-hemorrhagic) liquids were consistently observed (80%) in calves that survived for longer intervals (Fig. 4D). There was cellular debris inside the lung parenchyma (Fig. 4D,E) and thrombi in the pulmonary arteries (Fig. 4F). Although all newborn calves were treated with a surfactant, antibiotics and anti-inflammatory compounds, they had evidence of pulmonary hypertension and aseptic pneumonia. In addition, there were frequently signs of increased vascular resistance or high blood pressure in the pulmonary vasculature (Fig. 4F).

The heart had an average gross weight of 570 g (range, 137 to 743 g). However, there was no apparent association of heart weight and either body weight or survival rate (Table 1). In contrast, heart weight varied markedly among calves with similar body weight. For instance, the largest clone (No. 7, 62 kg) had a heart that weighted 743 g, whereas in another clone (No. 9, 55 kg), the heart weighed only 291 g. Regardless, the smallest heart was present in the smallest calf (Table 1). Severe heart defects were common. Calves that died soon after birth usually had two or more malformations, whereas those that survived longer had less severe cardiac problems. There was a wide range of alterations of the heart structure and the great vessels, approximately in 60% of the calves. In addition to previously reported malformations, there were hematic cysts in the tricuspid and mitral valves in all calves (Fig. 2E,3E); histologically these were pseudo-cystic spaces filled with blood (Fig. 2F). Persistence of the foramen ovale (Figs. 2G,3F) and defects of the ventricular septum, as well as patent ductus arteriosus (Fig. 2H), were also detected.

**Figure 5:**
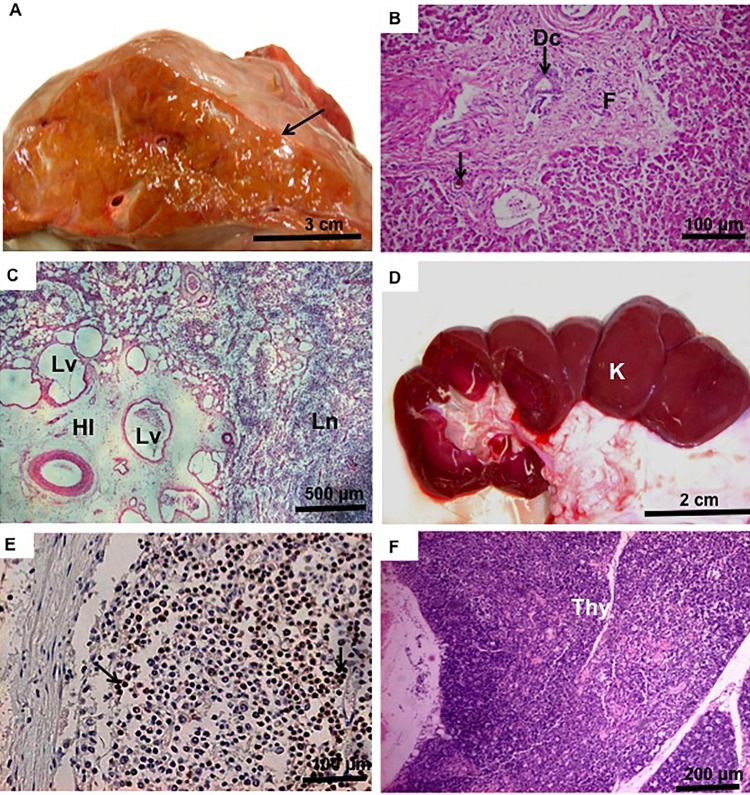
Details regarding internal organs of cloned bovine fetuses. **(A):** Overview of the liver with congestion in clone No. 13 that survive 3 d; note the yellowish color and irregular capsule (arrow). **(B):** Histology of the liver showing centrolobular fibrosis (F), bile cysts (arrow) and bile ducts (Dc). **(C):** Lymph node with severe edema, enlarged hillus (HI) including enlarged lymphatic vessels (Lv), and lymph nodes (Ln). **(D):** Kidney (K) with mild congestion. **(E)**: Immunohistochemistry for CD3 showing detection of T-lymphocytes (arrow) in the lymph nodes. **(F)**: Thymus (Thy) immature with only a few Hassal corpuscles.

The weight of the livers differed more than the other organs, with 420% variation among individuals without a clear association to body weight or survival rate (except that the smallest liver was in the smallest calf; Table 1). Most livers were yellowish, indicating degeneration, with mild to moderate hepatic congestion (more pronounced in those that survived longer), dilation of blood vessels, and areas of periportal necrosis (Fig. 5A,B). Peri-hepatic lymphadenomegaly was present in all calves (Fig. 5C). The kidneys seemed to be largely unaffected. However, in clones that survived longer, progressive congestion (Fig. 5D) and tubular degeneration were present, indicating circulatory insufficiencies. Most calves had enlarged lymph nodes, whereas some had a hypoplastic thymus (Fig. 5E). In one calf, the thymic parenchyma and the separation between the cortical and medullary areas was absent and Hassall’s corpuscles were not developed (Fig. 5F), indicating incomplete thymic maturation.

## Discussion

In the present study, bovine clones that survived until the neonatal period differed quantitatively and qualitatively from their *in vivo*-derived counterparts. Alterations affected several internal organs (including heart, lung and liver) and were likely associated with vascular disorders initiated during the early intrauterine phase. In that regard, the use of invasive ART, namely cloning by nuclear transfer, caused epigenetic modifications, resulting in disrupted organogenesis and posnatal problems, including vascular disorders, in cattle [45].

Cloned calves had a large range of body weights that were not clearly related to weight of internal organs or survival time. Nearly all calves were larger than normal or had outright gigantism [25,33]. In that regard, the calf that weighed approximately 62 kg at birth was larger than the maximum of 58.6 kg previously reported [25]. Also, we had one extremely lightweight individual (13 kg), much less than the 44 kg given as a minimal expected birth weight [25]. Based on the very small fetus with severe, but probably not lethal pathological features, we inferred that dwarfism may be an occasional effect of cloning. The dwarf fetus had a severe cardiac malformation, whereas such problems were not related to gigantism. Pathological data on other cloned animals including sheep, swine and mice [35–37,46] were consisted with the present findings in cloned bovine fetuses. For instance, cloned lambs suffered from the large offspring syndrome [47]; detailed analyses detected major cardiovascular, pulmonary, hepatic, and renal abnormalities, in addition to musculoskeletal issues.

Consistent with former studies [25,28,32,33], vascular disorders were common and had severe consequences. It was noteworthy that clones with a very short life span had multiple, severe alterations of the heart. Clones that died soon after birth had right ventricular dilatation with bilateral thickening of the ventricular wall, an increased diameter of the pulmonary artery and a patent ductus arteriosus [25]. Furthermore, Garry et al. [32] necropsied a newborn with a round and dilated heart. In addition, in the present study, a persistent foramen ovale and hematic cysts in the atrioventricular valves were common. We concluded that the affected individuals likely died due to circulatory system disorders. Furthermore, alterations in other internal organs seemed to be functionally related to vascular problems and hypertrophic cardiomyopathy. There are apparently no previous reports of biometric data relating size of cloned animals nor internal organs at the necropsy. In our results, there was a 442% variation in heart weight, in association with malformations that have apparently not been previously described. In cloned mice that had early death, although no clinical data were reported, there were consistently signs of cardiovascular alterations at necropsy, resembling congestive heart failure with hepatic and pulmonary consequences [36]. Other organs like the thymus had huge variations, and hemo-lymphatic alterations were described (e.g. thymus hypoplasia) [29]. This developmental problem, which can cause immune system insufficiency in adulthood, has also been described in adult cattle [30].

Our data supported former reports of pulmonary hypertension, lesions, edema and pleural effusions [25,28,32], in addition to capillary congestion of the alveolar septa and pulmonary thrombosis causing hemodynamic disturbances. These alterations likely inhibited complete alveolar expansion and explained the pulmonary insufficiency that contributed to the low postnatal survival rate of cloned calves [11,12,14,25,28,32,33]. Hepatic congestion, lymphadenomegaly and thymic hypoplasia may also be associated with heart and vascular diseases, especially the hypertrophy of the left ventricle that affects venous return from the abdominal organs. Related problems, such as hypoplasia of the thymus, have been reported [29].

Finally, alterations associated with the vascular system were evident in the intrauterine phase. In that regard, morphology of the heart, lungs and liver were altered in bovine embryos and fetuses derived from NT pregnancies, compared to pregnancies derived from other techniques [24]. Also, the placenta has been reported to have an unusual structure and vasculature, including high numbers of microplacentomes with dilated vessel architecture and loose fetomaternal contact in most areas, in addition to extensive areas of extravasated blood and edema [20,22,23], which likely reduced the efficiency of placental system. Placentomegalomy and increased diameter of umbilical vessels [22,34] may represent compensating mechanisms, which would be associated with fetal malformations, e.g. gigantisms of the fetal liver. However, in the present study, the developing vasculature in the chorioallantoic membrane was usually normal. In sum, we inferred that epigenetic modifications caused by the cloning process most likely caused vascular developmental disturbances (starting during early gestation) that affected differentiation and function of the placenta, as well as various internal organs. Some authors have related vascular and heart defects to hyper-expression of various genes, e.g. VEGF, BMP4, PCAF, FGF10, and Xist [49]. Indeed, VEGF expression differed between placentas of cloned versus non-cloned bovines calves at term [45]. Finally, the present results provided the rationale to determine expression of genes related to vascular development. Finally, that research on farm and laboratory animals has identified severe alterations associated with cloning procedures; future applications of such techniques in human beings are clearly not warranted.

## Conclusions

Although there was developmental pathology in a variety of organs in cloned cattle, we inferred that they had a common source in disturbances of the vascular system that occurred during early pregnancy. Perhaps, vascular developmental problems resulted as the primary or key alterations due to epigenetic modifications caused by cloning.
